# Introgression of mountain hare (*Lepus timidus*) mitochondrial DNA into wild brown hares (*Lepus europaeus*) in Denmark

**DOI:** 10.1186/1472-6785-6-17

**Published:** 2006-11-15

**Authors:** Tina Fredsted, Trine Wincentz, Palle Villesen

**Affiliations:** 1 Department of Ecology and Genetics, Institute of Biological Sciences, University of Aarhus, Ny Munkegade, building 1540, DK-8000 Aarhus C, Denmark; 2 National Environmental Research Institute, Dept. of Wildlife Ecology and Biodiversity, Grenaavej 14, DK-8410 Ronde, Denmark; 3 BiRC – Bioinformatics Research Center, University of Aarhus, H.-Guldbergs Gade 10, Building 1090, DK-8000 Aarhus C, Denmark

## Abstract

**Background:**

In Europe the mountain hare (*Lepus timidus*) exists in Great Britain, Norway, Sweden, Finland, parts of the Alps and in Eastern Europe, but not in Denmark. Interspecific hybridization has been demonstrated between native Swedish mountain hares and introduced brown hares (*Lepus europaeus*). During the data collection in a study concerning Danish brown hares we identified 16 hares with a single very divergent haplotype.

**Results:**

Phylogenetic analysis shows that the divergent Danish haplotype is most closely related to the Swedish mountain hare. The frequency of *Lepus timidus *mtDNA haplotype in the Eastern Danish hare populations is estimated to 6%.

**Conclusion:**

In contrast to what is known, the Danish hare populations are not pure *L. europaeus *populations but include introgressed brown hares with Swedish *L. timidus *mtDNA. The most probable explanation of this is natural migration or translocation of introgressed brown hares from Sweden. The impurity of hare populations has implications for conservation and population genetics.

## Background

The brown hare (*Lepus europaeus *Pallas, 1778) is widely distributed throughout Europe, up to 60°N, in Asia Minor and probably south to Israel. It is a popular game animal and its range has expanded to the east both by natural dispersion and by translocations to central and far-east Siberia [1]. Worldwide it has also been imported to many other countries, outside its natural distribution range, e.g. South and North America, Australia and New Zealand.

The brown hare is common all over Denmark and is the only *Lepus *species that exists in the country. Since 1960 the hare population has declined drastically in Europe and in Denmark. The primary cause of the decline is habitat changes in relation to agricultural intensification, whereas effects of climatic changes and predator abundance have increased by loss of year-round access to high-quality food and cover [2]. Additional factors like predators (especially foxes), birds of prey and traffic may have influenced the population locally [3]. In Denmark this is reflected by a large drop in annual number of hares shot from more than 400,000 before 1960 to 67,600 in 2004/2005 [4].

The general range of the mountain hare (*Lepus timidus *Linnaeus, 1758) covers much of the Palaearctic. In Europe it exists in Great Britain, Norway, Sweden, Finland, parts of the Alps and in Eastern Europe but it does not exist in Denmark [5]. During the late nineteenth century the brown hare was introduced in southern Sweden as a game animal [6]. In Scandinavia the mountain hare populations have been retreating northwards since the introduction of the brown hare, presumably as a result of a gradual competitive exclusion by the latter [7]. Mountain hares supposedly colonized Scandinavia through repeated immigration waves over two different post-glacial colonization routes; one from the south and one from the northeast [8].

Given the sympatric distribution of several hare species, e.g. in Sweden, Italy, and Spain, a number of studies focused on the possibility of interspecific hybridization. The first evidence of interspecific hybridization in hares concerns *L. timidus *and *L. europaeus *in Sweden [9-11] where unidirectional introgression of the native *L. timidus *mtDNA occurs in the introduced *L. europaeus*. However, introgression of mitochondrial DNA into other hare species has been demonstrated, e.g. the introgression of *L. timidus *mtDNA into *L. granatensis*, *L. europaeus *and *L. castroviejoi *in northern Iberia [12] and introgression of foreign mtDNA is likely to occur in several hare species from Asia [13]. These findings have lead to the conclusion that the genetic integrity of many European species largely depends on differences in behaviour and ecology which, at best, offer semi-permeable isolation [14]. In captivity, mountain hare females mate with brown hare males and produce viable offspring, whereas the reverse crosses do not happen spontaneously but can be performed successfully with insemination [15]. The F_1 _hybrids are morphological intermediates between the two species [16].

During the data analysis in a study concerning Danish brown hares we identified 16 hares with a very divergent haplotype. Here we show that these 16 individuals from the Danish wild brown hare populations carry mountain hare mtDNA haplotypes.

## Results

**Figure 1:**
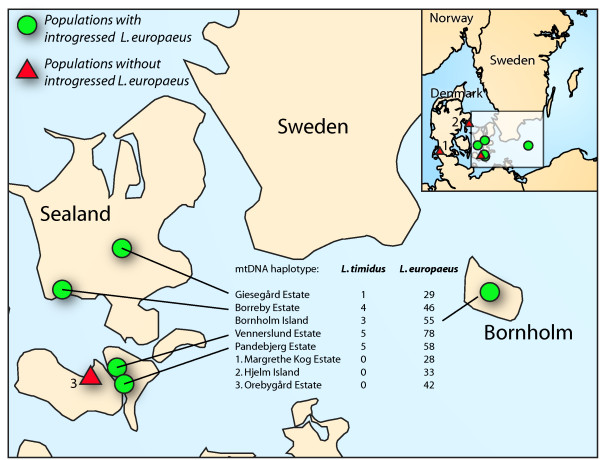
Location and frequency of the *Lepus timidus *haplotype in Denmark. *Lepus timidus *was found in five (large map) of the eight (small map) sampling locations.

During alignment of 385 hare sequences, 16 sequences (6 males, 10 females) were clearly different from the rest and collapsed into a single haplotype. The 16 individuals were obtained from five of the eight populations sampled, and these five populations are all located on islands in the eastern part of Denmark (Figure 1). The frequency of sampled individuals with *L. timidus *mtDNA in those five populations averaged 6% (a crude estimate across all eight populations is 4.16%, Figure 1).

**Figure 2:**
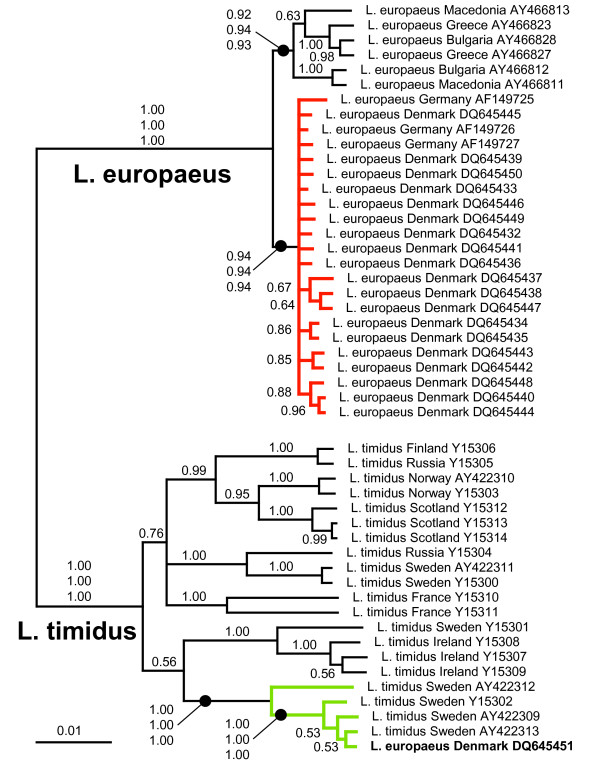
Bayesian phylogeny of 370 bp from the mitochondrial D-loop of 20 *Lepus timidus *and 28 *Lepus europaeus *haplotypes from Europe, including the 19 Danish *Lepus europaeus *haplotypes, and the divergent Danish haplotype. The numbers show nodal support, given as posterior probabilities (for selected nodes, posterior probabilities from three independent runs are shown).

A BLAST search against GenBank with the divergent haplotype mtDNA returned *L. timidus *as the best hit (99% similarity), but not a single *L. europaeus *sequence in the first 50 hits. The subsequent phylogenetic analysis (Figure 2) revealed that the divergent Danish haplotype is grouped with *L. timidus *samples from across Europe (100% posterior probability in all three independent runs) and most closely related to the Swedish *L. timidus *haplotypes (100% posterior probability in all three independent runs, green clade in figure 2). The other Danish haplotypes included in the phylogenetic analysis, the 19 *L. europaeus *haplotypes, are grouped with the *L. europaeus *clade as expected, and they are most closely related to German haplotypes (100% posterior probability from three independent runs, red clade in figure 2). Hence, the Danish haplotypes clearly originate from two different species.

## Discussion

The results show the presence of mountain hare mtDNA in Danish brown hare populations. From mtDNA alone we cannot decide whether these 16 individuals are true mountain hares or introgressed brown hares. This would require further analysis using nuclear gene sequences (e.g. *transferrin*) (there are no differential diagnostic microsatellite loci for the two species [17]). However, given a) the frequency of the *L. timidus *haplotype where it is present (6% in the five populations), b) that *L. timidus *is not reported to be a native breeding species in Denmark [5], and c) that the two species are able to hybridize [9], these individuals most likely represent introgressed brown hares.

The most likely origins of introgression are; 1) natural migration of mountain hares from Sweden followed by introgression, or 2) natural migration or translocation of introgressed brown hares from Sweden. However, since the documented distribution of mountain hares in Sweden does not include southern Sweden [9,10], natural migration of mountain hares is less probable than introduction of introgressed brown hares (carrying mountain hare mtDNA) from Sweden.

The fact that The Baltic Sea occasionally freezes over in very severe winters facilitates natural migration of introgressed brown hares from southern Sweden across the sea barrier to the Eastern Danish islands (Figure 1). This is also supported by the results from the genetic analyses (Figure 2).

During the mid 1980'ies a network of hare breeding facilities was established in Denmark, and it was initiated with imported hares primarily from Italy, France, Hungary and Sweden [18]. No combined record is kept of the amount and origin of hares imported to Denmark. In 1993, when farming hares were prohibited in Denmark, there were 100 hare-farms in Denmark, with an annual export of up to 5,500 hares [19]. As approximately 15% of Swedish *L. europaeus *specimens from sympatric areas carried transmitted *L. timidus *mtDNA [10], it is possible that phenotypic brown hare specimens imported from Sweden for breeding purposes have carried the *L. timidus *mtDNA and later escaped to the natural hare population.

The detection of only one haplotype in the 16 individuals shows low mtDNA diversity in the total population of hybrids in Denmark. The geographical distribution of the 16 intogressed brown hares (Figure 1) and the lack of haplotype diversity support a common origin in southern Sweden.

The implications of introgressed brown hares in the Danish hare populations are currently unclear. It has been suggested that hares with an alien mtDNA have a lowered fitness as a result of a functional incompatibility between the cytoplasmatic mitochondrial genes and the cell *nucleus *[10]. In opposition to this, due to its observed high frequency in brown hares (93%) Melo-Ferreira *et al*. [20] suggested, that the ancient *L. timidus *mtDNA observed in the Iberian Peninsular might have some selective advantage depending on the nuclear background.

Thulin *et al*. [17] raised the question as to whether any 'pure' population of brown hares exists anywhere. Our findings give further support to this statement. This has important consequences for conservation and population genetics, e.g. problematic definition of management units, unclear fitness effects, genetically mixed populations and the inclusion of nuclear markers will be necessary for future hare studies.

## Conclusion

Contrary to what is known, the Danish hare populations are not pure *L. europaeus *populations but include introgressed brown hares with Swedish *L. timidus *mtDNA. The most probable explanation is natural migration or translocation of introgressed brown hares from Sweden. The impurity of hare populations has implications for conservation and population genetics.

## Methods

### Sample collection

During 2003–2006 The National Environmental Research Institute of Denmark collected tissue samples from shot, wild hares at eight locations in Denmark (Figure 1). The hares were autopsied and several organs and muscle tissue samples were taken out for later analyses.

### Sequencing

DNA was extracted from hare muscle tissue using a slight modification of the Chelex protocol [21]. The tissue was placed into 1.5 mL Eppendorf tubes containing 200 μl of 20% Chelex resin solution (BIO-Rad, Hercules, CA, USA). Tubes were vortexed briefly, boiled at 100°C for 20 min in a heating block, centrifuged 3 min at 13000 rpm and stored at minus 20°C.

A 494 bp fragment of the mtDNA D-loop (control region) was amplified via PCR, using the mammalian control region primers L15997 5'-CACCATTAGCACCCAAAGCT-3' located in the tRNA gene and H16498 5'-CCTGAAGTAGGAACCAGATG-3' [22]. PCR was performed in a total volume of 10 μl and contained 1 μl buffer (1.5 mM MgCl_2_), 1.6 μl dNTP (1.25 mM of A, C, G, T, respectively), 0.5 μl of each primer (10 pmol/μl), 0.1 μl *Taq *polymerase (Amersham Pharmacia Biotech), topped up with distilled water to 9 μl and 1 μl template DNA (40–60 ng/μl) was added. Cycling conditions were 94°C for 3 min, and 35 cycles of 94°C for 30 s, 50°C for 40 s and 72°C for 60 s and a final extension step of 72°C for 7 min. Sequencing was conducted under BigDye™ terminator cycling conditions, the reacted products purified using ethanol precipitation and run using an Automatic Sequencer ABI 3730xl. Both strands were sequenced in all samples.

### Haplotype editing and collapsing

Sequences were edited using Bioedit version 7.0.0 [23]. Identical haplotypes among the 385 sequences were found using DNAcollapser [24].

### Phylogenetic analyses

Nine *L. europaeus *and 20 *L. timidus *sequences were downloaded from GenBank. Sequences with geographical information were selected to represent haplotypes from all over Europe and Russia. Together with the 19 Danish *L. europaeus *and the divergent haplotype, these 49 sequences (table 1) were aligned using Muscle [25] and cropped to the shortest sequence (370 bp). Phylogenies and nodal support were estimated using MrBayes, version 3.0b4 [26] under a Bayesian framework [27,28] using a general time reversible substitution model (GTR + γ) and the coalescent branch length model. Bayesian analysis was initiated with random starting trees, run for 5 × 10^6 ^generations, and the Markov chain was sampled every 1000 generations. Model parameters were estimated directly from the data and three independent replicates were conducted to avoid entrapment in local optima [29]. The initial 1,250 trees were discarded as "burn-in" and the remaining 3,750 trees were used to estimate nodal support as posterior probabilities.

## Authors' contributions

TF carried out all the lab-work, edited and analyzed the sequence data and drafted the manuscript. PV carried out the phylogenetic analysis, prepared the figures and drafted the manuscript. TW organized the samples and drafted the manuscript. All authors have read and approved the final manuscript.

## Figures and Tables

**Table 1:** Sampling localities and accession numbers of D-loop sequences used in the phylogenetic analysis (figure 2).

Species	Sampling locality	Accession #
*L. europaeus*	Bulgaria	AY466812
*L. europaeus*	Bulgaria	AY466828
*L. europaeus*	Macedonia	AY466811
*L. europaeus*	Macedonia	AY466813
*L. europaeus*	Greece	AY466823
*L. europaeus*	Greece	AY466827
*L. europaeus*	Germany	AF149725
*L. europaeus*	Germany	AF149726
*L. europaeus*	Germany	AF149727
*L. timidus*	Sweden, Grimso	AY422312
*L. timidus*	Sweden, Kalix	AY422311
*L. timidus*	Norway, Ringebu	AY422310
*L. timidus*	Sweden, Salsta	AY422313
*L. timidus*	Sweden, Vaxvik	AY422309
*L. timidus*	Finland	Y15306
*L. timidus*	France	Y15310
*L. timidus*	France	Y15311
*L. timidus*	Ireland	Y15307
*L. timidus*	Ireland	Y15308
*L. timidus*	Ireland	Y15309
*L. timidus*	Norway	Y15303
*L. timidus*	Russia	Y15304
*L. timidus*	Russia	Y15305
*L. timidus*	Scotland	Y15312
*L. timidus*	Scotland	Y15313
*L. timidus*	Scotland	Y15314
*L. timidus*	Sweden	Y15300
*L. timidus*	Sweden	Y15301
*L. timidus*	Sweden	Y15302
*L. europaeus*	Denmark	DQ645432
*L. europaeus*	Denmark	DQ645433
*L. europaeus*	Denmark	DQ645434
*L. europaeus*	Denmark	DQ645435
*L. europaeus*	Denmark	DQ645436
*L. europaeus*	Denmark	DQ645437
*L. europaeus*	Denmark	DQ645438
*L. europaeus*	Denmark	DQ645439
*L. europaeus*	Denmark	DQ645440
*L. europaeus*	Denmark	DQ645441
*L. europaeus*	Denmark	DQ645442
*L. europaeus*	Denmark	DQ645443
*L. europaeus*	Denmark	DQ645444
*L. europaeus*	Denmark	DQ645445
*L. europaeus*	Denmark	DQ645446
*L. europaeus*	Denmark	DQ645447
*L. europaeus*	Denmark	DQ645448
*L. europaeus*	Denmark	DQ645449
*L. europaeus*	Denmark	DQ645450

***L. europaeus, introgressed***	Denmark	DQ645451
